# Cell size and cell cycle progression: the cyclin-dependent kinase link in green algae

**DOI:** 10.1093/jxb/erz008

**Published:** 2019-02-04

**Authors:** Angharad R Jones, Walter Dewitte

**Affiliations:** School of Biosciences, Cardiff University, United Kingdom

**Keywords:** Cyclin-dependent kinase (CDK) activity, *Desmodesmus quadricauda*, DNA replication, light/dark intervals, nuclear and cellular division, protein, RNA, starch accumulation, temperature

## Abstract

This article comments on the following paper:

**Zachleder V, Ivanov I, Vítová M, and Bišová K.** 2019. Effects of cyclin-dependent kinase activity on the coordination of growth and the cell cycle in green algae at different temperatures. Journal of Experimental Botany 70, 845–858.

**The coordination of cell growth and cell division is a fundamental problem in biology. The experiments with unicellular algae of Zachleder *et al.* (2019) show that up-regulation of cyclin-dependent kinase (CDK) activity can drive cell progression even when biosynthesis is reduced, indicating that the main link between cell growth and cell division is CDK activity rather than overall biosynthesis. Understanding this coupling between cellular growth and CDK activity will be important not just in these algae, but also in higher plants.**

In most eukaryotes, replication of the genome during S-phase and segregation of that DNA into daughter cells during M-phase is separated by two gap phases. During the first gap phase (G_1_), which separates the end of M-phase and the relaunching of DNA synthesis, cells commit to re-entering the cell division cycle. During the second gap phase (G_2_) important quality controls are made to prevent premature separation of daughter cells. Concurrent with this mitotic cell cycle is a cellular growth cycle. Whilst a close coordination between cell division and cell growth is required to maintain a sustainable cell size in future generations of daughter cells, there is flexibility in this relationship and cells can respond in both cell size at division and cell cycle length depending on the experimental species, the conditions and environmental context. The challenge now lies in understanding the molecular mechanisms that underpin this flexibility.

## Cell division and cell growth, a flexible relationship

Much of the pioneering work on the relationship between cell growth and cell division was done in the fission yeast *Schizosaccharomyces pombe*. Cell cycle progression is driven by the action of cyclin-dependant kinases (CDKs), which function in feed-forward loops that act at the key cell cycle transitions (Novak *et al.*, 1998; Zhao *et al.*, 2012; Barr *et al.*, 2016; Perez-Hidalgo and Moreno, 2016). These loops reinforce the activity of the CDKs and degrade cell cycle regulators, creating switch-like behaviours that prevent the cycle from running backwards. In yeast, there is a single cyclin-dependent kinase, CDK1. Cell cycle control is achieved by a setting a lower threshold for CDK1 activity for G_1_/S transition compared to G_2_/M transition (Coudreuse and Nurse, 2010). Kinase activity is reset after mitosis, and gradually increases afterwards through G_1_, S and G_2_. Nutritional control influences the proportional lengths of G_1_ and G_2_ (Rubio *et al.*, 2018) and the cellular growth rate. High levels of nutrients shorten G_1_ and lengthen G_2_, while lower levels of nutrients confer a longer G_1_ and relatively shorter G_2_. Rather than being an indirect relationship, in *S. pombe* this process is tightly controlled by the TOR-mediated PP2A activity mechanism that controls the activity of CDK1 and allows cell division at smaller sizes under starvation conditions (Perez-Hidalgo and Moreno, 2016). In higher plants and algae the G_1_/S transition requires CDKA activity while G_2_/M activity is regulated by the action of CDKA and CDKB kinases. Given that there are two classes of kinases involved in the cycle, one could speculate that this allows for more complexity in its regulation.

In higher plants cells do not migrate as they are attached to each other with a cell plate, so the local context is instrumental in promoting or constraining growth and division. Despite this, cell division still appears to be closely linked to cell growth. Higher plant cell size is increased under favourable growth conditions, and in floral primordia where cells are larger cell cycle length is reduced (Jones *et al.*, 2017) suggesting that increased capacity for biosynthesis, including CDK biosynthesis, supports faster growth and faster cell cycles. Interfering with kinase activity at the G_1_/S or G_2_/M transition results in an extended G_1_ or G_2_, respectively, but as the total cell cycle length is maintained a mechanism whereby cellular growth is coupled to cell cycle progression either via CDK biosynthesis or thresholding was proposed (Jones *et al.*, 2017).

The unicellular green alga *Chlamydomonas reinhardtii* proliferates by a multi-fission cycle. In the *C. reinhardtii* division cycle, cells must attain two or more separate commitment points during a long G_1_ growth phase. These large cells then undergo a repeated sequence of S- and M-phases followed by synchronous release of the daughters. In parallel to the mechanisms identified in higher plants, CDKG1 accumulates during cell growth in G_1_ and, together with its CYCD binding partner, inactivates the Retinoblastoma tumour suppressor protein (RB) to allow the G1/S transition. Although *C. reinhardtii cdkg* mutants are not delayed in reaching commitment, they do become larger during G_1_ than wild-type cells in line with observations of Arabidopsis *cycd3* mutants. As in Arabidopsis, there was no increase in overall cell cycle length of the *C. reinhardtii cdkg* mutant, hence a function for CDKG1 as a sizer was proposed that would link growth to division by acting on the RB pathway (Li *et al.*, 2016). In Arabidopsis CDKGs have been implicated in salt stress, meiotic stability and splicing (Zheng *et al.*, 2014; Ma *et al.*, 2015; Cavallari *et al.*, 2018), but a sizer role has not been attributed.

## Uncoupling cell division from biosynthesis

The report by Zachleder *et al.* (2019) investigates this relationship in the unicellular green alga *Desmodesmus quadricauda,* which shares CDKA and CDKB kinases with higher plants. In this system, cells undergo multiple rounds of DNA synthesis and nuclear division, before finally partitioning the cytoplasm in a single cytokinesis step. In contrast to the *C. reinhardtii* cycle, the multiple synthesis phases may overlap and daughter cells can remain attached to one another in a multicellular structure known as a coenobium. Each synthesis phase follows a growth phase and is dependent on the cell passing a commitment point. Progression is also limited by environmental conditions; light intensity restricts photosynthesis and the production of RNA and proteins, while temperature affects the overall duration of the division cycle. Since the number and frequency of reproductive sequences is correlated with growth conditions it has been supposed that attainment of the commitment point is related to reaching a critical cell size. Here Zachleder and colleagues uncover an unusual relationship between cell size and cell cycle length that suggests that division may be independent of cell growth in this system.

In comparison to the systems mentioned previously, in which small cells are expected to have long cell cycles, Zachleder and colleagues observed that although cells grown at higher temperatures were smaller than cells grown at lower temperatures, they were still able to cycle faster than the cold-grown cells. This suggests that the biosynthetic capacity of the cell is not the major determinant of the increase of CDK activity in this system. In fact, cells grown at higher temperatures accumulated a higher level of CDK activity than cold-grown cells, despite producing a lower total amount of protein. The independence of biosynthesis and increased CDK activity were further demonstrated by transferring cells grown at 20 °C to 30 °C. After changing temperature, cells were kept in the dark so that no further RNA or protein synthesis took place. Despite the absence of protein synthesis, these cells showed an increase in CDK activity and continued to divide. This is significant as it confirms that CDK activity is primarily dependent on temperature and is sufficient to drive cell cycle progression even in the absence of cell growth. Understanding how temperature or other signals that control CDK activity are integrated into cell cycle regulation might therefore uncover points of regulation that could be exploited in higher plants to uncouple growth and division.

To separate growth from division at the molecular level, the production of CDK activity needs to be uncoupled from the biosynthetic constraints operating within the cell. This could be achieved by dedicating a larger proportion of the cell’s protein synthetic capacity to the production of CDK activators, but is more likely the result of post-translational modifications that increase activation of the existing CDK pool (Box 1). For example, mathematical modelling demonstrates that rapid switch-like activation of CDK can be achieved through the rapid removal of inhibitory phosphorylations and degradation of inhibitors (Novak *et al.*, 1998; Zhao *et al.*, 2012; Barr *et al.*, 2016; Perez-Hidalgo and Moreno, 2016). This kind of activation also explains the observation that yeast cells that have passed the commitment point will complete division even if environmental conditions subsequently become less favourable. In the algal experiments this was not the case; cells moved from high to low temperatures did not continue to divide, meaning that it is possible to pause the cell cycle even after commitment. This extra flexibility, possibly conferred by the multiple kinases active at G_1_/S and G_2_/M, is likely to be beneficial given the increased investment of resources required for multiple nuclear divisions.

Box 1. **Regulation of CDK activity**

The increase of Cyclin-Dependent Kinase (CDK) activity under elevated temperature introduces the question of the underlying mechanism. Is this a consequence of increased enzymatic activity at this elevated temperature, or is it achieved by a different regulation at the molecular level? CDK activity is regulated at several levels. Without interacting CYCLIN (CYC) the CDKs are inactive, so the concentration of cyclins and CDKs as well as their affinity comes into play. The concentration is determined by the production and degradation rate. Cyclins are degraded by the 26S proteasome, and indeed a well-controlled turnover is essential for mitotic progression, but little is known as to how cyclin or CDK turnover is under environmental control. Activation of the CYCLIN/CDK complex requires post-translational activation. Inhibitory phosphorylations—conferred by kinases such as WEE1—which block the catalytic site can be removed by phosphatases or bypassed by boosting the kinase activity by elevating the expression of other CDKs such as the CDKB family. CDK-activating kinases provide T-loop stabilization so that the active site becomes accessible. The interaction with the ICK/KRP and SIAMESE inhibitors is a third level of control. KRP levels are under hormonal control and these are crucial for controlling cell numbers in tissues during plant development. But the interaction with these factors might also promote the formation of the CYCLIN/CDK complexes by providing a scaffold. Furthermore interaction with factors such as the Cyclin-dependent Kinase Subunits (CKS) seem to play a role in mitigating their activity or targeting in response to stress (Inze and DeVeylder, 2006).

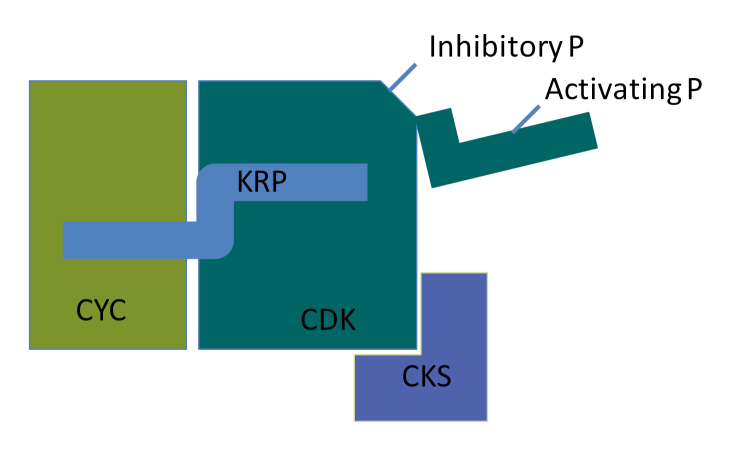

## Perspectives

Mechanisms that link cell size and cell cycle progression are thought to be central to creating cell size control. It will therefore be interesting to address how the uncoupling of growth and CDK activity in *D. quadricauda* affects cell size, and what role this may play in long-term survival of the organism. For example, is the elevation of CDK activity critical for this species to adapt to elevated temperatures? And, if so, is the ultimate target the size of individual cells or the overall coenobium structure? Which mechanism underpins this? Is it a consequence of the enzymatic properties of the CDK or does it involve posttranslational modifications or degradation of inhibitors? Answering these questions may identify a new link between environmental signals and CDK activity as well as providing an interesting comparison between unicellular organisms and higher plants in which such trade-offs between cell number and cell size are relevant during development.

In conclusion, the observations presented by Zachleder *et al.* (2019) suggest that activation of CDK activity can drive division and overcome suboptimal conditions for cellular growth in these algal species. This raises the question as to how cellular growth is coupled to CDK activity in both these algae and higher plants.
